# Association between DDT or its byproducts and T2DM: a systematic review and meta-analysis

**DOI:** 10.3389/fendo.2025.1634292

**Published:** 2025-10-06

**Authors:** Weijian Tian, Shaohui Bai, Ting Xie, Haolu Zha, Zhouning Yan, Shengze Zhang, Nan Wu, Jianhui Yuan, Huanle Luo, Qian Xie, Ying Jiang

**Affiliations:** ^1^ Shenzhen Nanshan Center for Disease Control and Prevention, Shenzhen, China; ^2^ School of Public Health (Shenzhen), Shenzhen Key Laboratory of Pathogenic Microbes and Biosafety, Sun Yat-sen University, Shenzhen, China; ^3^ Key Laboratory of Tropical Disease Control (Sun Yat-sen University), Ministry of Education, Guangzhou, China

**Keywords:** DDT, byproducts, meta-analysis, T2DM, quantitative evidence

## Abstract

**Background:**

Currently, type 2 diabetes mellitus (T2DM) is among the fastest-growing global health emergencies of the century. Emerging evidence from epidemiological studies suggests a potential positive association between exposure to dichlorodiphenyltrichloroethane (DDT) or its byproducts and an increasing risk of T2DM.

**Objective:**

We conducted a systematic review and meta-analysis to quantify the association between DDT or its byproducts and T2DM. Additionally, we aimed to identify the sources of heterogeneity contributing to the inconsistency of the results.

**Methods:**

Data analysis: assess the quality of the included studies using the Risk Of Bias In Non-randomized Studies of Exposure tool, determine the source of heterogeneity using subgroup analysis, sensitivity analysis and meta regression model based on a fixed-effects model, and analyze the publication bias using funnel plots, Egger’s test, and Begg’s test.

**Results:**

DDT and its byproducts were associated with the risk of developing T2DM (total OR: 1.13, 95% CI: 1.08-1.15, I^2^ = 40%). Subgroup analysis stratified by biomarkers showed a stronger association between p,p′-DDE and T2DM (OR = 1.13, 95% CI: 1.09-1.17, I²= 58.5%). The results of the funnel plot, Egger’s test, and Begg’s test showed publication bias and small study effect in studies included in the analysis (p<0.05), but the influence on the results was smaller.

**Conclusions:**

The systematic review and meta-analysis offer quantifiable proof of a positive correlation between exposure to DDT or its byproducts and a higher risk of developing T2DM.

**Systematic Review Registration:**

https://inplasy.com, identifier INPLASY20258004.

## 1 Introduction

Dichlorodiphenyltrichloroethane (DDT) or 2,2-bis (4-chlorophenyl)-1,1,1-trichloroethane is an organochlorine pesticide used extensively since the 1940s to combat vector-borne illnesses like malaria and typhoid fever, as well as agricultural pests (1). It produces several byproducts, including dichlorodiphenyldichloroethylene (DDD), dichlorodiphenyldichloroethane (DDE), *p,p*′-DDT, *o,p*′-DDT, *p,p*′-DDD, *o,p*′-DDD, *p,p*′-DDE, and *o,p*′-DDE. DDT exists in nature as both dissolved and particulate forms and shares characteristics with other organochlorine compounds, including lipophilicity, stability, persistence, bio-accumulation, and biomagnification (2). These properties allow it to infiltrate surface water sources, degrade water quality, and accumulate in ecosystems through the food chain (3). The half-life period of *p,p*′-DDT and *p,p*′-DDE is respectively 7 years (4) and 10 years in human serum (5). Humans can accumulate DDT through direct contact with or ingestion of contaminated water or food, posing health risks (6). However, it remains detectable at high concentrations in regions where DDT use has ceased, making it an ongoing global environmental concern (2). In response to these concerns, some affluent countries began phasing out DDT in agriculture in 1972 due to its increasing environmental impact and the Stockholm Convention was adopted by approximately 152 countries in 2001, establishing a global ban on persistent organic pollutants (7). However, in 2006, the World Health Organization (WHO) authorized the use of DDT for indoor residual spraying in countries where malaria, dengue, yellow fever, and so on remain a significant health issue.

Type 2 diabetes mellitus (T2DM), also known as adult-onset diabetes, represents 90%-95% of all diabetes cases. According to the latest estimates from the International Diabetes Federation, it is projected that one in eight adults (approximately 783 million) will have diabetes, with over 90% of these cases being T2DM by 2045. This condition is one of the fastest-growing global health emergencies of this century (8, 9). Numerous risk factors, such as environmental toxins including DDT and its byproducts, have an impact on T2DM. Epidemiological studies in many groups have demonstrated a stronger link between the risk of T2DM and exposure to organochlorine insecticides (10, 11), among which *p,p*′-DDT may play an important role in the etiology of T2DM (12, 13). Exposure to *p,p′*-DDT and *p,p′*-DDE may affect glucose metabolism, cause insulin resistance, interfere with body thermogenesis, and affect the regulation of lipids and glucose, according to experimental animal research and *in vitro* and *in vivo* evidence (14–16).

The primary pathophysiological mechanism underlying T2DM involves impaired insulin signaling despite normal pancreatic β-cell insulin secretion. This condition, known as insulin resistance, manifests as the inability to effectively suppress hepatic glucose production and regulate blood glucose levels. At the molecular level, insulin resistance results from either reduced insulin receptor density or post-receptor signaling defects in insulin-sensitive tissues (17). A hallmark manifestation of insulin resistance in T2DM involves impaired GLUT4 translocation to the plasma membrane in the absence of insulin signaling, resulting in deficient glucose uptake. Additionally, diminished activity of downstream insulin receptor effector enzymes (e.g., PI3K/Akt pathway components) contributes significantly to the pathogenesis of T2DM by disrupting normal glucose homeostasis (18). Research also shows that obesity caused by excessive nutritional intake, oxidative stress, and so on is an important cause of insulin resistance (19, 20). Furthermore, accumulating epidemiological data implicate DDT exposure as a potential risk factor for T2DM, likely mediated through its deleterious effects on insulin signaling pathways. Emerging research demonstrates a significant association between DDT and its metabolites and impaired pancreatic function. DDT mainly enters the human body through contaminated food, water, and air and is distributed to fatty tissue, the liver, kidneys, pancreas, and other organs through the bloodstream and its liposoluble properties (21). Current literature indicates that serum, plasma, and whole blood concentrations of DDT and its primary metabolite, DDE, typically range from 1 to 500 nM in human populations. However, existing toxicological data lack established safe exposure thresholds (NOAELs) or direct evidence regarding pancreatic toxicity in humans (22–24). The most frequently mentioned topic was the impact of DDT and its metabolites on protein expression in human pancreatic beta cells (24). For example, chronic exposure of p,p′-DDT to pancreatic β cells has been found to result in reduced protein expression of genes associated with hyperglycemic stress response (24). Persistent organic pollutants, especially organochlorine pesticides, have been shown to significantly inhibit insulin action and insulin-induced gene 1 (Insig-1) and Lpin1 (16). Further studies have shown that DDT may influence the endoplasmic reticulum stress response associated with insulin resistance in diabetes. Additionally, DDT increases the expression levels of PERK and IRE1a, molecules associated with the activation of the unfolded protein response (UPR) in the molecular axis of T2DM (25–28). There are also studies showing that DDT exposure can induce inflammation and oxidative stress to inhibit the activity of insulin receptor substrate (IRS)-2, thereby promoting insulin resistance, disrupting the insulin signaling pathway, and disrupting glucose homeostasis (29, 30). Multiple prospective cohort studies have demonstrated a robust association between DDT exposure and the incidence of diabetes mellitus or insulin resistance. These findings align with prior epidemiological research utilizing serum organochlorine pesticide concentrations as biomarkers. Furthermore, accumulating evidence indicates that persistent organic pollutants (POPs) serve as significant predictors for the development of future insulin resistance and/or T2DM (11, 31, 32).

Although various studies have shown that organochlorine pesticides (including DDT and its byproducts) may be an environmental risk factor for T2DM, there are still some epidemiological studies that reveal that DDT or its byproducts may not be a risk factor for T2DM development (33–35). The reasons for discrepancies in these results could stem from various factors, including variations in bias control procedures, exposure determination techniques, and data accessibility. Previous studies have used meta-analysis to evaluate the association between exposure to organochlorine pollutants and the risk of developing T2DM in populations. However, the results imply that the association between exposure to DDT or its byproducts and the development of T2DM is smaller or negligible (36–38). At the same time, these studies did not apply proper methodologies to examine the impact of factors like small study effect and publication bias on the robustness of the results. The disparities in these studies’ findings could be attributed to a number of factors, including a dearth of high-caliber research, thorough analysis techniques, thorough biomarker classification, and other factors that affect how results are integrated. Therefore, we compiled the benefits and drawbacks of previous studies to offer more thorough and trustworthy data for the examination of the connection between DDT or its byproducts and T2DM. Consequently, by applying standard guidelines for the inclusion of as wide a range of pertinent studies as possible to execute a meta-analysis, our integrated assessment of the evidence gathered from cohort studies and case–control studies of the potential adverse effects of DDT and its byproducts on T2DM will provide favorable evidence for the public health decision-making process. Our combined findings, which were obtained by using a more thorough meta-analysis technique, demonstrated a favorable correlation between DDT and T2DM.

## 2 Materials and methods

### 2.1 Protocol and guidance

This study was conducted according to the Preferred Reporting Items for Systematic Reviews and Meta-Analyses (PRISMA). This systematic review and meta-analysis have been registered on the INPLASY website with the following details: Registration number: INPLASY202580049.

DOI number: 10.37766/inplasy2025.8.0049.

### 2.2 Study’s inclusion and exclusion criteria

#### 2.2.1 Inclusion criteria

The literature must include cohort or case–control studies that adhere to the PECOS principles (Population, Exposure, Comparability, Outcome, and Study Design). Study subjects must be selected based on their exposure to DDT and its byproducts, as well as whether they have been diagnosed with T2DM (gold standard for confirmed diagnosis of T2DM: fasting blood glucose ≥7.0 mmol/L, 2-h glucose ≥11.1 mmol/L after glucose tolerance test or HbA1 ≥6.5%). Outcome indicators should be obtained through standardized measurements of biological specimens, such as serum or adipose tissue.

#### 2.2.2 Exclusion criteria

The following are the exclusion criteria: non-English, such as reviews, expert commentaries, conference papers, cross-sectional studies, and case studies; control populations with conditions other than T2DM, such as other renal diseases or insulin resistance; animal studies; studies with insufficient information on outcome effect indicators or those relying on environmental data or other indirect methods (questionnaires, lifestyle assessments) and studies where the full text is unavailable.

### 2.3 Data sources and search strategies

We identified epidemiological evidence of the association between exposure to DDT or its byproducts and T2DM by searching Chinese and English databases such as PubMed, Web of Science, and Embase. The included literature was published for the full database period. Database search strategies: (2,2-bis (4-chlorophenyl)-1,1,1-trichloroethane or dichlorodiphenyltrichloroethane or dichlorodiphenyldichloroethylene or dichlorodiphenyldichloroethane or DDT or DDTs or DDE or DDD or *p,p*′-DDT or *o,p*′-DDT or *p,p*′-DDD or *o,p*′-DDD or *p,p*′-DDE or *o,p*′-DDE) and (type 2 diabetes mellitus or T2DM or T2D) (**Supplementary Table 3**).

### 2.4 Literature screening

The retrieved literature was independently screened by two authors after removing duplicates by title and abstract to assess their relevance to our identified research questions. Subsequently, the article authors obtained the full text of these documents and extracted data entered for those that met the inclusion and exclusion criteria. Differences in authorship of the retrieved screened literature were adjusted for consistency.

### 2.5 Data extraction

Data from the included literature were independently extracted into an excel spreadsheet, capturing the following information: study type, title of study, first author, year of publication, journal and country of publication, cohort name, sample size and group, participants’ gender, ethnicity, and age, source and method of inclusion, type of exposure, endpoints and their ascertainment, effect indicators and their confidence intervals, adjusting variables, and length of follow-up time.

### 2.6 Quality of evidence and risk of bias assessment

The Risk Of Bias In Non-randomized Studies - of Exposure model is suitable for the risk of bias in observational epidemiological studies assessing the effects of environmental exposures on health outcomes. The risk of bias of the studies in the literature that we ultimately included was examined independently by two authors using this model (39). The tool overs seven evaluation domains (risk of bias due to confounding, risk of bias arising from measurement of the exposure, risk of bias in selection of participants into the study (or into the analysis), risk of bias due to post-exposure interventions, risk of bias due to missing data, risk of bias arising from measurement of the outcome, and risk of bias in selection of the reported result). These results were then integrated into the Grading of Recommendations Assessment, Development, and Evaluation evidence grading framework (40).

### 2.7 Data synthesis

We adopt Stata 17.0 for statistical analysis. We performed risk ratio (RR) or odds ratio (OR) and their associated 95% confidence interval (95% CI) to assess the risk of association between DDT or its byproducts exposure and T2DM and considered a *P* value less than 0.05 to be statistically significant. We used *I^2^* to assess their heterogeneity (when significant heterogeneity was not present (*I^2^* < 50%), we used fixed effects models; we used random-effects models when significant heterogeneity was present (*I^2^* ≥ 50%). We performed forest plot, meta regression, and sensitivity analysis to evaluate the existence of heterogeneity, the robustness, and reliability of the combined results. Furthermore, we utilized the funnel plot, Egger’s test, and Begg’s test to assess the possibility of publication bias and small study effect.

## 3 Results

### 3.1 Eligible studies and study characteristics

**Figure 1:**
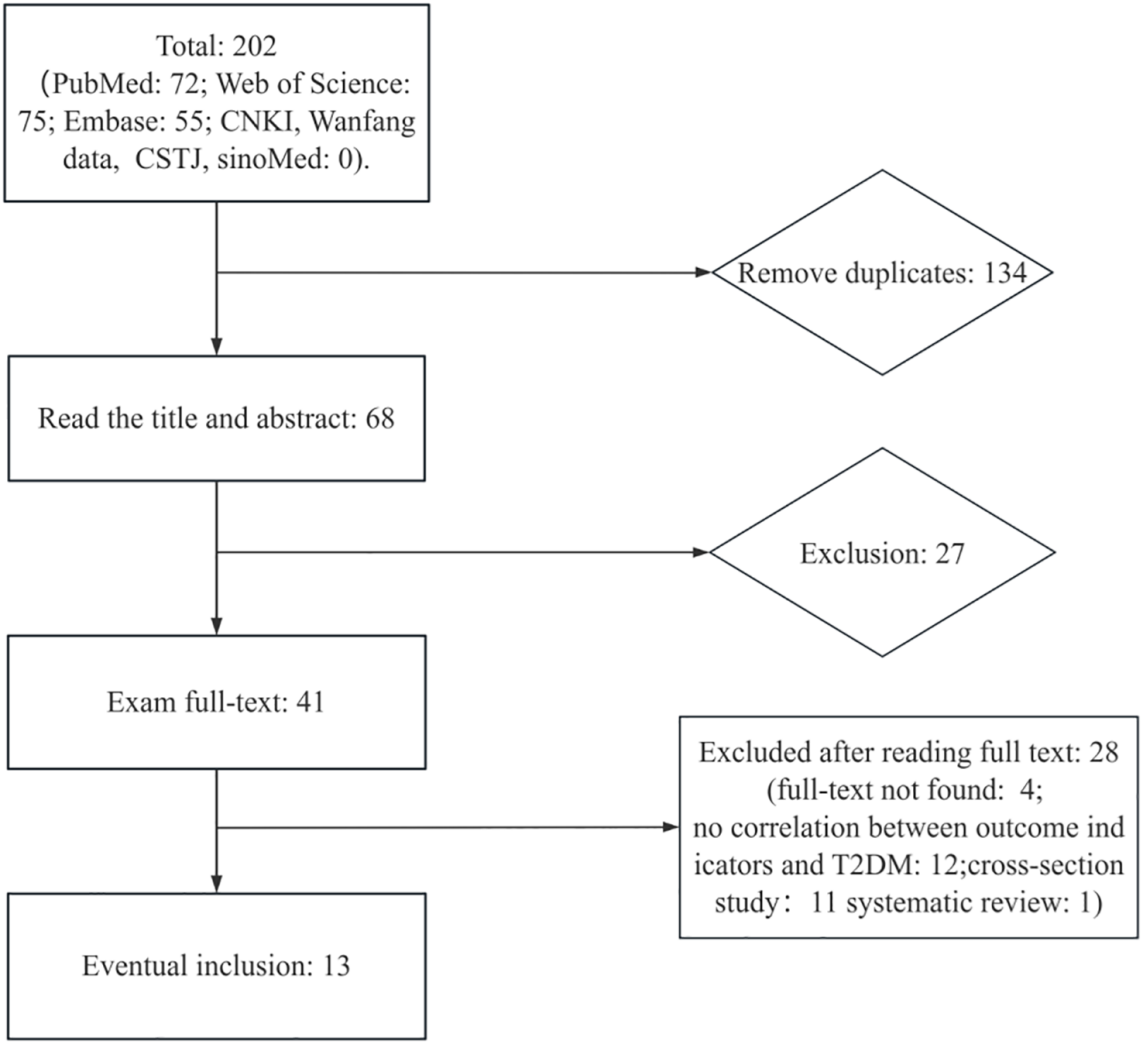
The study search and selection process.

We identified 202 references potentially relevant to the research question through searches across multiple databases for the final meta-analysis (**Figure 1**). After removing 134 duplicate references, the remaining 68 were screened based on title and abstract relevance, leading to 41 references selected for full-text review. Among these, 28 were excluded after full-text review: 4 lacked access to full text, 12 had weak correlations with T2DM outcome metrics, 11 were cross-section studies, and 1 was a systematic review. Ultimately, 13 articles were included for data extraction. Study characteristics are summarized in **Supplementary Table 1**. Among these 13 articles included, 9 were case–control studies, 2 were nested case–control studies, 1 was a case–cohort study, and only 1 was a prospective cohort study. The studies were conducted between 2007 and 2024. Three studies were conducted in Sweden, two in Korea, one in the United States, and four in China, with the remainder conducted in India, Algeria, France, and Norway. These studies used DDT or its byproducts (DDD, DDE, *p,p*′-DDT, *o,p*′-DDT, *p,p*′-DDD, *o,p*′-DDD, *p,p*′-DDE, and *o,p*′-DDE) as a biomarker. Various models were employed to assess RR or OR, with 95% CI adjusted for confounders such as age, gender, educational attainment, smoking status, drinking status, physical activity, body mass index, and other factors. Samples included serum, blood (plasma), adipose tissue, and other types. Several studies showed a dose–response relationship between DDE or its byproducts and the risk of T2DM.

### 3.2 Quality of study

The quality evaluation results for the 13 studies are presented in **Supplementary Table 2**. According to the assessment, most of the included epidemiological studies showed a low risk of bias or some concerns across the seven bias risk domains. However, three to four studies exhibited a high risk of bias due to missing data and selection of the reported results. It was important to note that these studies were a design where controlling for bias was inherently challenging. Overall, the studies we included were of sufficient quality for analysis.

### 3.3 Main analysis and subgroup analysis

**Figure 2:**
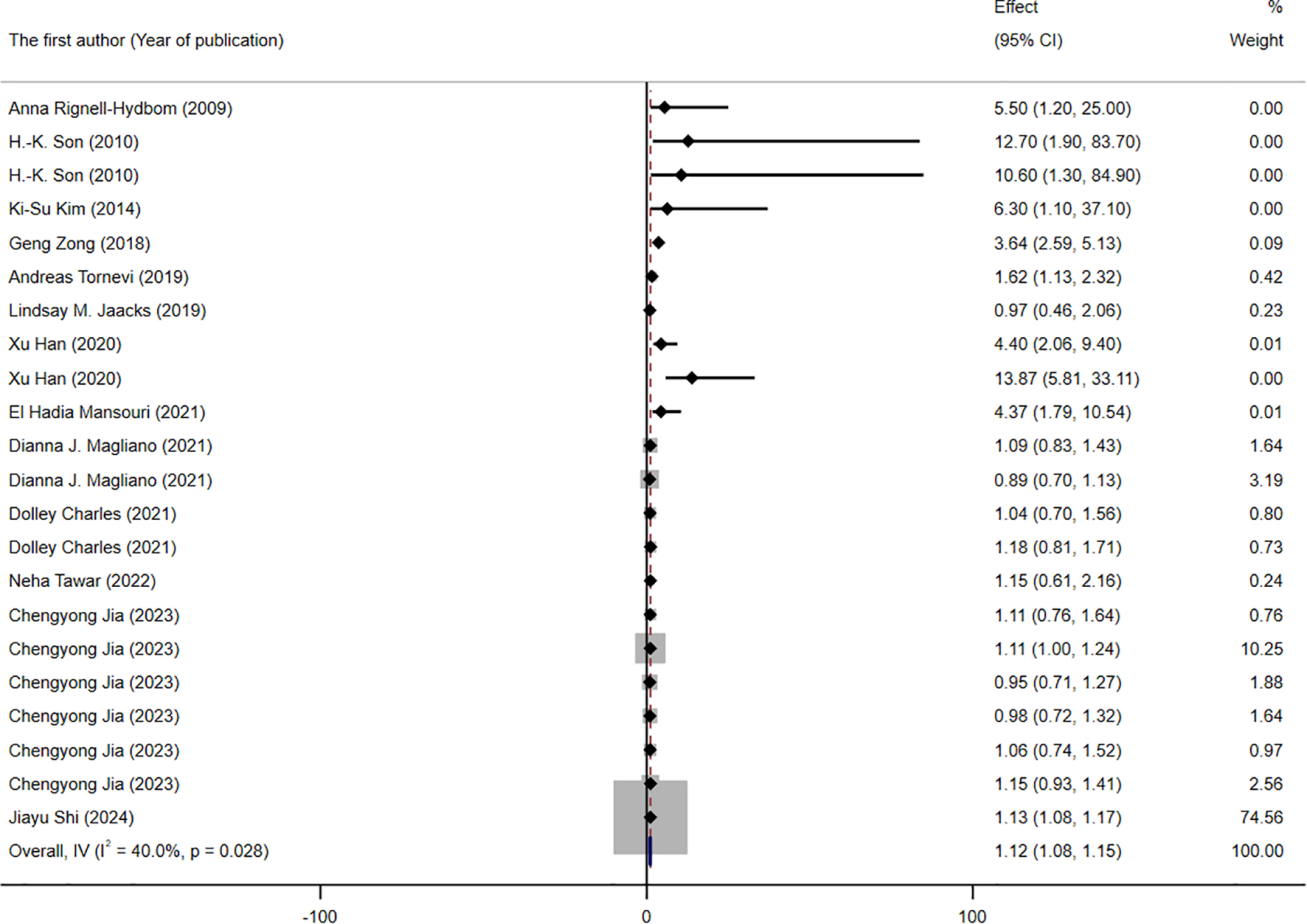
Forest plot of the association between exposure to DDT or its byproducts with T2DM, from human prospective studies and case–control studies.

A total of 13 studies, contributing 22 RR or OR estimates that met the inclusion criteria, were included in the analysis. Among these, 13 studies demonstrated a positive association between DDT or its byproducts and the prevalence of T2DM (**Figure 2**). The combined OR estimate, calculated using a fixed-effects model, was 1.12 (95% CI: 1.08-1.15, *I^2^* = 40%). This result is relatively robust. Forest plots displayed the weight of each study, with the study (41) having the highest weight at 74.56%. Subsequently, we also conducted subgroup meta-analysis to further explore and analyze the sources of this heterogeneity.

**Figure 3:**
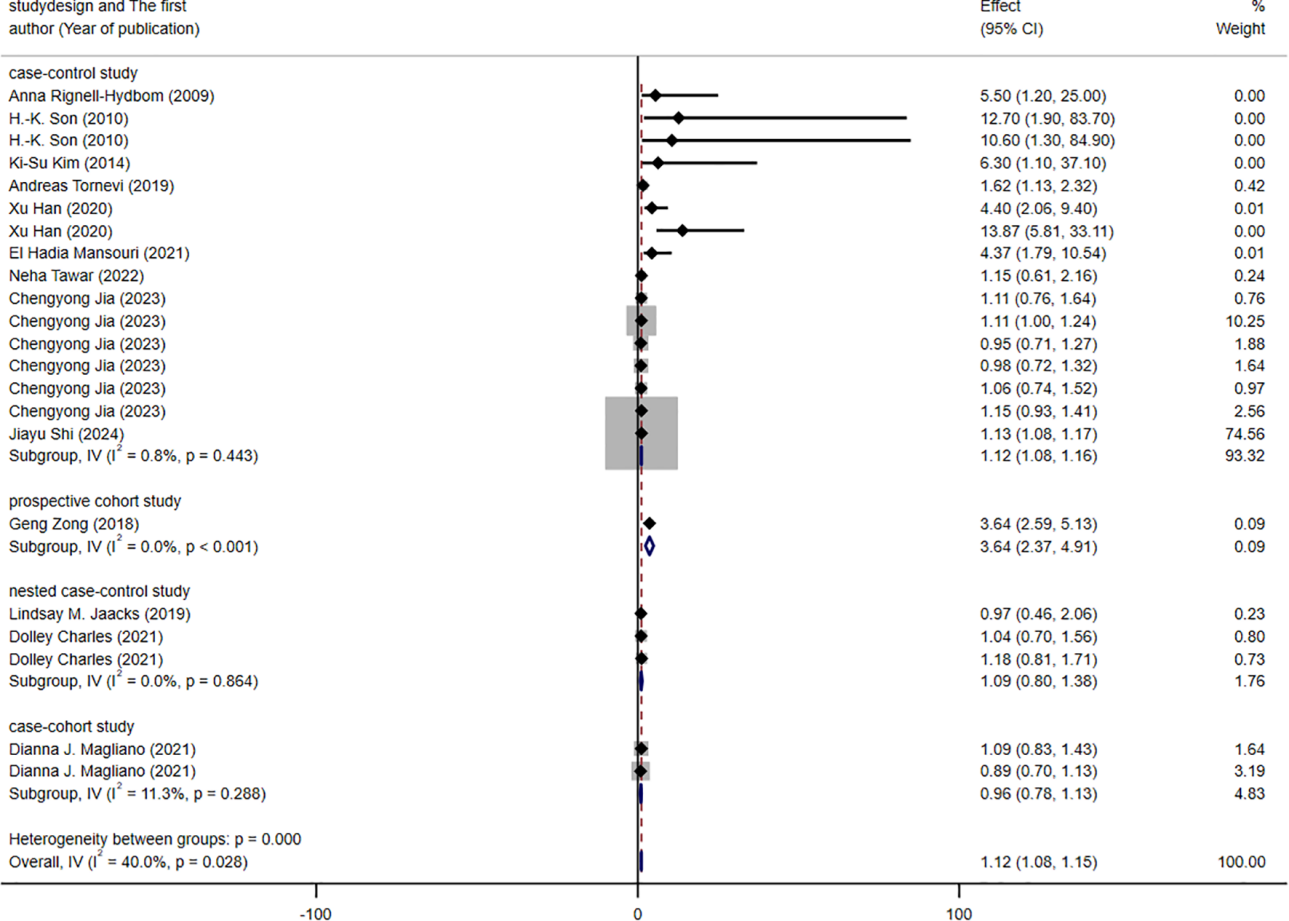
Forest plot of the association between exposure to DDT or its byproducts with T2DM, from human prospective studies and case–control studies, stratified by study design.

**Figure 4:**
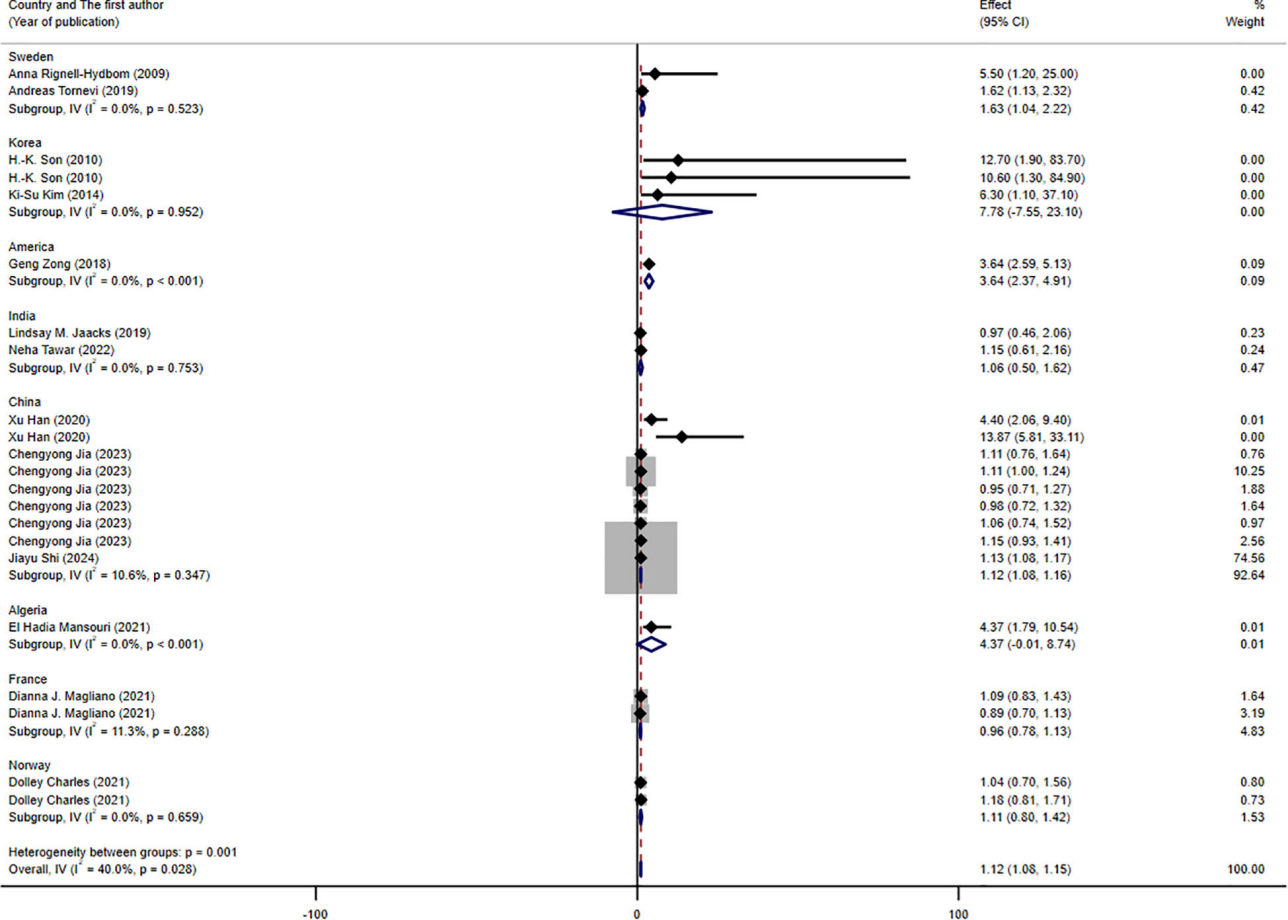
Forest plot of the association between exposure to DDT or its byproducts with T2DM, from human prospective studies and case–control studies, stratified by country.

**Figure 5:**
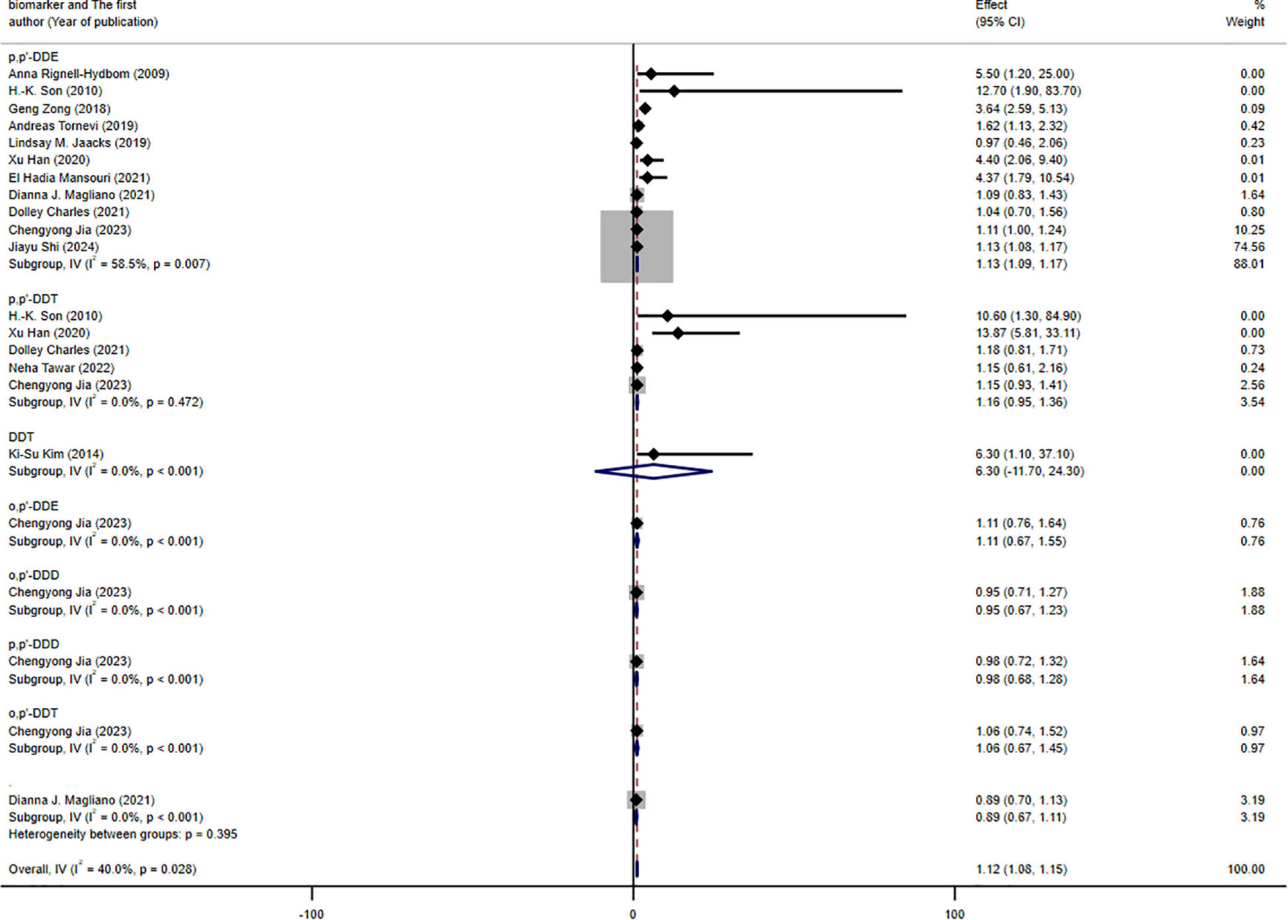
Forest plot of the association between exposure to DDT or its byproducts with T2DM, from human prospective studies and case–control studies, stratified by biomarker.

The results of the subgroup analysis are presented in **Figures 3**–**5**. We performed subgroup analysis based on study design, country, and biomarker to identify sources of heterogeneity. The study design and country-based stratification indicated that they were not the sources of this heterogeneity. Case–control study and prospective cohort study all indicated positive associations between DDT or its byproducts and T2DM (OR = 1.12, 95% CI: 1.08-1.16, *I²* = 0.8%, OR = 3.64, 95% CI: 2.37-4.91, *I²* = 0%, respectively). Positive associations between DDT or its byproducts and T2DM were observed in Sweden, America, and China, with the following results: Sweden (OR = 1.63, 95% CI: 1.04-2.22, *I²* = 4.0%), America (OR = 3.64, 95% CI: 2.37-4.91), *I²* = 0%), and China (OR = 1.12, 95% CI: 1.08-1.16, *I²* = 10.6%). In the biomarker-based stratification, *p,p*′-DDE was identified as the main source of heterogeneity (*I²* = 58.5%). The results indicated a positive association between *p,p*′-DDE and T2DM (OR = 1.13, 95% CI: 1.09-1.17, *I²* = 58.5%). Overall, the findings suggested a significant positive association between DDT or its byproducts and T2DM development.

### 3.4 Sensitivity analysis

Overall, the sensitivity analysis results indicated that the fixed effects model we used was reliable. The study (41) had the most significant impact on the consolidated effect estimate. However, the point estimate of the pooled effect size of the meta-analysis excluding this trial was within the 95% CI of the total pooled effect size. Similarly, all of the studies, the point estimates from the sensitivity analysis came within the 95% CI of the total combined effect size. The results of the sensitivity analysis showed that the results of the main analysis were robust in **Supplementary Figure 1**.

### 3.5 Meta regression

To further investigate the sources of heterogeneity, we conducted a meta-regression analysis, considering country, study design, and biomarker as concomitant variables. The results indicated that neither study design nor biomarker was a significant source of heterogeneity (*p* > 0.05), but it implied that the country was the origin of diversity as detailed in **Table 1**.

**Table 1:** DDT and its byproducts meta-regression with random effects—estimates of heterogeneity based on RR or OR effect sizes.

Covariate	Coef	SE	95% CI	*P*
Country	−0.90	0.36	−1.67 to −0.14	0.02
Study design	0.12	0.16	−0.20-0.45	0.44
Biomarker	−0.08	0.11	−0.32-0.16	0.50
Cons	3.53	0.91	1.62-5.44	0.001

Coef, coefficient; SE, standard error; Cons, constant.

### 3.6 Publication bias and small study effect

**Figure 6:**
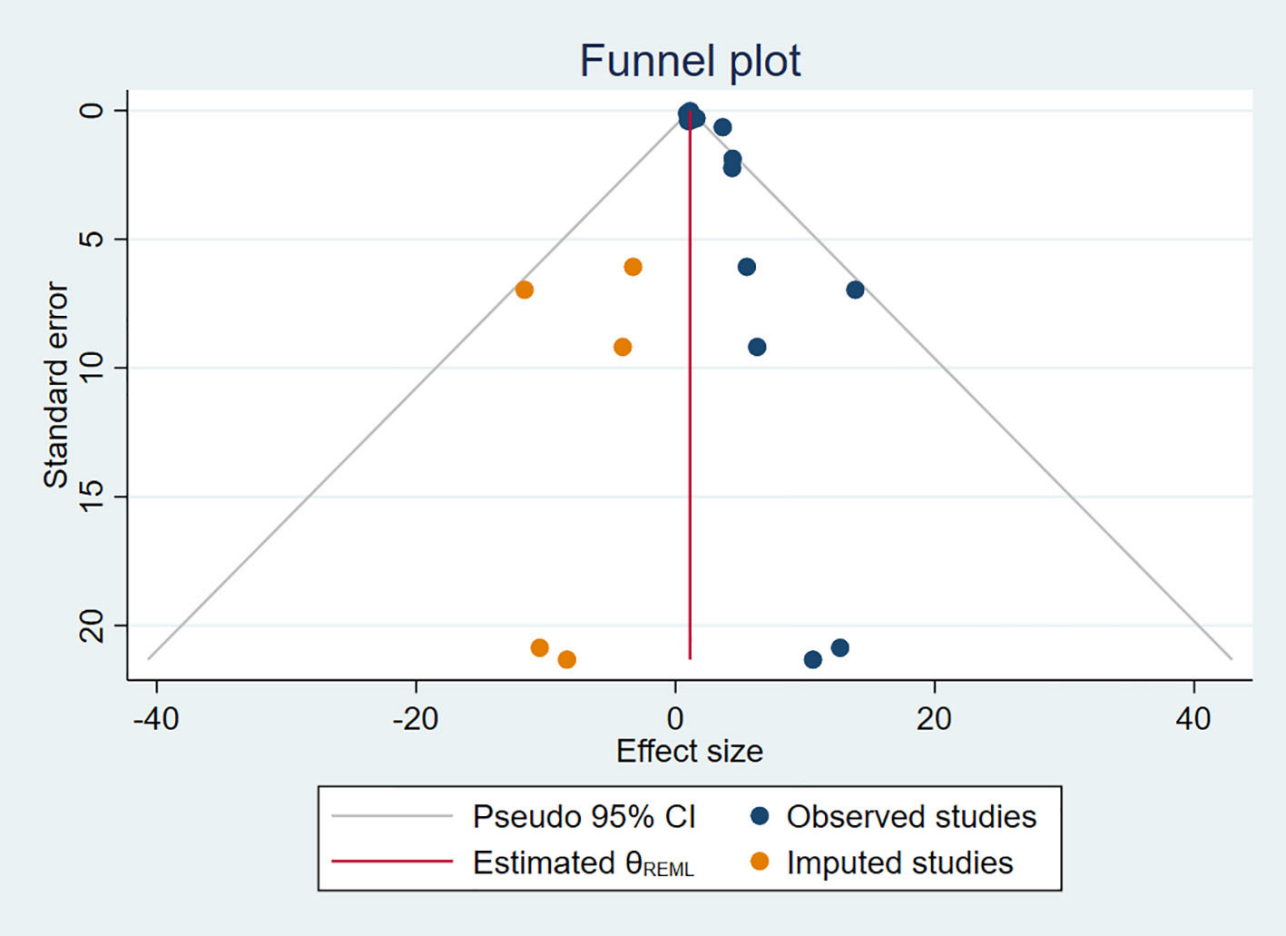
Trim and fill method for publication bias.

Additionally, we used a funnel plot to assess the publication bias and small study effect among studies with more than 10 data points, as shown in **Figure 6**. Visual inspection of the funnel plot suggested that there was evident publication bias and there could be a small study effect among the studies meeting the inclusion criteria. To further quantify and analyze whether this bias and small study effect existed or not, we conducted Egger’s test and Begg’s test. The results confirmed the presence of significant publication bias and small study effect (*p* < 0.05), as detailed in **Tables 2** and **3**. Therefore, we performed a trim and fill method of the funnel plot to estimate the influence of publication bias. This result showed that the association between DDT or its metabolites and T2DM may be overestimated due to the asymmetry of the funnel plot, but the effect is not significant as shown in **Table 4**. Sensitivity analysis showed that the small study effect was not significant, as shown in **Supplementary Figure 1**.

**Table 2:** Egger’s test^a^.

*β*	Z	SE	*P*
0.55	2.18	0.25	0.029

a The conversion of binary variable data is facilitated by “logOR”, selogOR.

**Table 3:** Begg’s test.

Kendall’s score	Z	SE	*P*
81.00	2.26	35.46	0.024

**Table 4:** Trim and fill method for public bias.

Studies	Exp(ES)	95% CI
Observed	3.052	2.937-3.171
Observed + imputed	3.051	2.936-3.171

Exp(ES): The conversion of binary variable data is facilitated by “gen logOR=log(_ES)”; “gen selogOR=_selogES”.

## 4 Discussion

DDT, a prototypical persistent organic pollutant (POP), was extensively utilized in agricultural and vector-borne disease control programs (notably malaria eradication) during the mid-20th century. However, mounting toxicological evidence has since revealed concerns regarding its chronic health implications. Initial epidemiological investigations examining the DDT–T2DM association were predominantly limited to ecological study designs conducted during the 1980-1990s period. For example, researchers discovered correlations by comparing DDT usage data (such as pesticide consumption records) from different regions with macro trends in T2DM incidence rates (42, 43). Nevertheless, ecological study designs are inherently limited by the ecological fallacy, a well-documented methodological constraint that precludes causal inference. A case in point is the 1990s agricultural cohort analysis that identified a positive association between DDT exposure and T2DM incidence. However, this study design could not adequately control for potential confounding variables, including dietary patterns or genetic predisposition, which may have independently influenced diabetes risk (44). The 21st century witnessed a critical transition in environmental epidemiology, shifting from ecological to individual-level exposure assessment (42). This methodological advancement addressed a key limitation: the need for precise quantification of both pollutant body burdens and metabolic outcomes. A landmark 2006 study by Lee et al. (22) analyzed NHANES data, revealing that serum DDE (the primary DDT metabolite) concentrations >75th percentile conferred 1.5-2.0-fold higher T2DM risk (p<0.01). Subsequent prospective cohorts, including the Agricultural Health Study (AHS), validated this association while adjusting for confounders (BMI, smoking, and occupational co-exposures) (45). This shift made the evidence more reliable and revealed a dose–response relationship. It also paved the way for mechanistic studies. For example, DDT may act as an endocrine disruptor, affecting insulin signaling pathways and β-cell function (42, 46).

Up to now, a large number of studies about the association between organochlorine pollutant (including DDT and its byproducts) exposure and the risk of T2DM have been published, but few cohort studies were available (47). Furthermore, there are still contradictory epidemiological results from these studies that point to a relationship between exposure to DDT or its metabolites and the risk of T2DM (48). These findings can be attributed to variations in the study population chosen, geographic locations, sources of DDT and its byproducts exposure, and so on (49, 50). Similarly, scarcely any systematic reviews and meta-analysis were used to conclude the association between exposure to DDT or its byproducts and the risk of T2DM, especially excluding Fakhri’s study (38). Regretfully, subgroup analysis, publication bias, and small study effect detection were not done in this study. Instead, the main analysis was the only analysis done for the various study types. At the same time, the quality of the included studies was not assessed using conventional quality evaluation instruments. Comprehensively summarizing strengths and weaknesses of previous studies, we firstly conducted a comprehensive systematic review and meta-analysis special for the DDT or its byproducts with T2DM. Numerous perspectives were taken into consideration when analyzing and interpreting the results.

In contrast to previous studies (37, 38, 47), the meta-analysis we have conducted on the relationship between DDT or its byproducts and T2DM leaves much to be desired. Firstly, the included literature was published for the full database period. To guarantee the specificity of the included studies and their relevance to the study’s goal, we limited the search terms (DDT and all its byproducts). Subsequently, we assessed the quality of the included studies using the model to ensure high quality for meta-analysis. At the same time, only cohort and case–control studies were considered in order to guarantee the validity of the combined findings. Secondly, we investigated the size of the combined effects and the cause of heterogeneity using the main analysis, subgroup analysis, sensitivity analysis, and meta-regression model. The combined estimate of effect is 1.12 (95%CI, 1.08-1.15, *I^2^* = 40%, *p* < 0.05). The results of subgroup analysis and meta-regression showed that the biomarker (*p,p*′-DDE, *I^2^* = 58.5%, *p* < 0.05) or country(*p* < 0.05) was the source of heterogeneity in generating the meta-analysis, respectively. Positive associations between DDT or its byproducts and T2DM were observed in Sweden, America, and China, with the following results: Sweden (OR = 1.63, 95% CI: 1.04-2.22, *I²* = 4.0%), America (OR = 3.64, 95% CI: 2.37-4.91), *I²* = 0%), and China (OR = 1.12, 95% CI: 1.08-1.16, *I²* = 10.6%). This suggests that the moderate heterogeneity of our results may be due to the variety of by-products and proportions of country-related studies included. These results were different from those of previous studies. In contrast, we used a more robust fixed-effects model with less heterogeneity in the pooled effect size. At the same time, based on earlier research (38), sensitivity analysis and meta-regression analysis were added, and the results indicated that the small sample impact had minimal bearing on the total effect size. Last, we also quantified the presence of publication bias and small study effect by using the funnel plot, the Egger’s test, and the Begg’s test. Additionally, we employed trim and fill of the funnel plot for the first time to demonstrate the extent to which publication bias in our study affected the findings. The results after pruning showed that publication bias overestimated the relationship between DDT or its byproducts and T2DM, but the difference in this relationship was not too large, indicating that our results had good accuracy. Likewise, our study also has some limitations. First, non-Chinese or non-English literature was not included. Second, some important confounding factors were not included and analyzed. Third, we did not include cross-sectional studies for analysis due to methodological limitations. Fourth, there are still some important biases in the published studies that we were not able to balance statistically. Last, the effects of co-interaction cannot be analyzed by us.

Differences in the type of study design, type of exposure, country, methods of measuring exposure, etc., all lead to variations in the results of the association of DDT or its byproducts with T2DM and introduce heterogeneity in the meta-analysis. Therefore, addressing these sources of variation effectively requires a more thorough and scientific approach. Although we analyzed the presence of small study effect using meta-regression models. However, this model can only analyze linear effects and not the presence of non-linear effects. These potential non-linear effects can have a more significant impact on the results of the combined effects measures. Some participants may not have been completely unexposed, which may have underestimated the calculation of the true effect. Human beings are exposed to DDT and its byproducts (long-term low-dose) throughout their lifetime. In the meantime, these chemicals will accumulate in the adipose tissue and be released into the bloodstream due to lipophilicity, stability, persistence, bioaccumulation, biomagnification, and so on, thereby likely further disrupting various biological functions (3, 16, 51). It has also been suggested that DDT and its byproducts cause oxidative damage to the organism and thus cause T2DM (51). Due to the diversity of targets and factors affecting the action of DDT and its byproducts on living organisms, they cannot be considered to have a single mode of action (52).

Studies have also shown that organochlorine insecticides inhibit insulin action at low doses, but this effect disappears at high doses, suggesting that there may not be a clear dose–response relationship (16). The studies included in the analysis contained results on the effects of DDT and its derivatives on the pancreas. Pavlikova et al. analyzed the downregulation of four proteins (cytokeratin 8, cytokeratin 18, actin, and alpha-enolase) in pancreatic β cells exposed to sublethal concentrations of DDT and DDE (24). Tawar et al. further revealed that DDT interacts with host genes and plays a role in T2DM by detecting a positive correlation between endoplasmic reticulum stress markers in patients with diabetes who were exposed to DDT (53). Studies have also shown that DDT can induce oxidative stress and damage mitochondria, thereby disrupting insulin signaling pathways and affecting the pancreas, which can lead to insulin resistance (24, 29, 30, 54, 55). The high quality of the evidence included is ensured by our research, which sets strict inclusion and exclusion criteria and quality assessment standards. At the same time, we also referred to the excluded literature on the relationship between DDT and insulin resistance. We identified the association between DDT and T2DM by extracting, integrating, and analyzing information from high-quality studies. We then clarified the potential relationship between DDT and T2DM by integrating and discussing the relevant literature on DDT and insulin resistance, demonstrating how DDT may affect insulin function and contribute to the development of T2DM. The main mechanisms and dosage range of DDT and its metabolites affecting the occurrence of T2DM are shown in **Supplementary Table 4**.

There have been other studies suggesting that potential mechanisms for the relationship between organochlorine pesticide exposure and T2DM include binding to the various receptors (46, 56, 57). There could be a variety of ways that DDT and its byproducts affect T2DM. Determining or assessing the link between DDT and T2DM alone is inaccurate, leading to hidden bias in the combined effects. The relationship between DDT or its byproducts and T2DM needs to be carefully interpreted.

In summary, despite the influence of these reasons, our results can still suggest that DDT and its byproducts are indeed associated with an increased risk of T2DM. Although the effect of this correlation is not very large based on a small sample of the study, the impact of this small effect on a large population cannot be ignored. Perhaps some control measures for DDT and its byproducts will have unintended consequences for the health effects of T2DM in the population. At the same time, the adoption of some sound public health measures may also have large health economic benefits. These results also provide evidence support for further studies on DDT and its byproducts with the risk of T2DM.

## Data Availability

The original contributions presented in the study are included in the article/**Supplementary Material**. Further inquiries can be directed to the corresponding author/s.
