# Supplementary material for: Association between DDT or its byproducts and T2DM: a systematic review and meta-analysis
Source: Front Endocrinol (Lausanne). 2025 Oct 6;16:1634292. doi: 10.3389/fendo.2025.1634292 (PMC12536502; doi:10.3389/fendo.2025.1634292)
Supplement: Supplementary file 1 [file DataSheet1.docx]

1. **Supplementary Figure**

**Supplementary Figure 1. Sensitivity analysis.**

1. **Supplementary Tables**

**Supplementary Table 1. Study characters.**

| **Study design** | **Reference** | **Journal** | **Country** | **Controls/Cases** | **Age** | **Biomarker** | **Biologic speciman** | **RR or OR(95%CI)** |
| --- | --- | --- | --- | --- | --- | --- | --- | --- |
| case-control study | Anna Rignell-Hydbom2009 | PLOS ONE | Sweden | 371/371 | 50-59 | p,p'-DDE | serum | 5.50  (1.20-25.0) |
| case-control study | H.-K. Son2010 | Environment International | Korea | 40/40 | 48-73 | p,p'-DDE | serum | 12.70  (1.90-83.7) |
|  |  |  |  |  |  | p,p'-DDT |  | 10.60  (1.30-84.9) |
| case–control study | Ki-Su Kim2014 | Chemosphere | Korea | 25/25 | 61-74 | DDTs | visceral adipose tissue/subcutaneous adipose tissue | 6.30  (1.10-37.1)^a^ |
| prospective cohort study | Geng Zong2018 | Environment International | America | 793/793 | 25-42 | p,p'-DDE | blood | 3.64 (2.59-5.13)^a^ |
| case–control study | Andreas Tornevi2019 | Environmental Research | Sweden | 129/129 | 52-63 | p,p'-DDE | Blood plasma | 1.62 (1.13-2.32)^a,b^ |
| nested case-control study | Lindsay M. Jaacks2019 | Environment International | India | 323/193 | 25-56 | p,p'-DDE | Blood plasma | 0.97 (0.46-2.06)^a^ |
| case-control study | Xu Han2020 | Ecotoxicology and Environmental Safety | China | 158/158 | 25-74 | p,p'-DDE | serum | 4.40  (2.06-9.40)^a^ |
|  |  |  |  |  |  | p,p'-DDT |  | 13.87 (5.81-33.1)^a^ |
| case-control study | El Hadia Mansouri2021 | Chemosphere | Algeria | 181/180 | 27-73 | p,p'-DDE | blood sample | 4.37 (1.79-10.54)^a,c^ |
| case-cohort study | Dianna J. Magliano2021 | Diabetes & Metabolism | France | 583/200 | 30-65 | p,p'-DDE | serum | 1.09 (0.83-1.43) |
|  |  |  |  |  |  | p,p'-DDT |  | 0.89 (0.70-1.13) |

**^a^Different models adjusted by confounding, such as age, gender, BMI, educational attainment, smoking status, drinking status, physical activity, total cholesterol and triglycerides,and various compounds.**

**^b,c^Stratified by the BMI and ages respectively.**

**Supplementary Table 1. Cont.**

| **Study design** | **Reference** | **Journal** | **Country** | **Controls/Cases** | **Age** | **Biomarker** | **Biologic speciman** | **RR or OR(95%CI)** |
| --- | --- | --- | --- | --- | --- | --- | --- | --- |
| nested case-control study | Dolley Charles2021 | Environmental Research | Norway | 139/116 | 37-56 | p,p'-DDE | serum | 1.04 (0.70-1.56)^a^ |
|  |  |  |  |  |  | p,p'-DDT |  | 1.18 (0.81-1.71)^a^ |
| case–control study | Chengyong Jia2023 | Environmental Pollution | China | 1006/1006 | 55-71 | o,pʹ-DDE | blood | 1.11 (0.76-1.64)^a^ |
|  |  |  |  |  |  | p,pʹ-DDE |  | 1.11 (1.00-1.24)^a^ |
|  |  |  |  |  |  | o,pʹ-DDD |  | 0.95 (0.71-1.27)^a^ |
|  |  |  |  |  |  | p,pʹ-DDD |  | 0.98 (0.72-1.32)^a^ |
|  |  |  |  |  |  | o,pʹ-DDT |  | 1.06 (0.74-1.52)^a^ |
|  |  |  |  |  |  | p,pʹ-DDT |  | 1.15 (0.93-1.41)^a^ |
| case–control study | Jiayu Shi2024 | Environmental Pollution | China | 908/904 | 44-66 | p,pʹ-DDE | blood | 1.13 (1.08-1.17)^a^ |
| case–control study | Neha Tawar2022 | Frontiers in Endocrinology | India | 70/70 | 34-54 | p,p′-DDT | The visceral adipose tissue，blood | 1.15  (0.61-2.17)^a^ |

**^a^ Different models adjusted by confounding, such as age, gender, BMI, educational attainment, smoking status, drinking status, physical activity, total cholesterol and triglycerides,and various compounds.**

**^b,c^ Stratified by the BMI and ages respectively.**

**Supplementary Table 2. Estimate of study quality.**

| **Reference**  **Class of risk** | **Risk of bias due to confounding** | **Risk of bias arising from measurement of the exposure** | **Risk of bias in selection of participants into the study** | **Risk of bias due to post-exposure interventions** | **Risk of bias due to missing data** | **Risk of bias arising from measurement of outcomes** | **Risk of bias in selection of the reported result** |
| --- | --- | --- | --- | --- | --- | --- | --- |
| Anna Rignell-Hydbom2009 | Low risk | Low risk | Some concerns | Low risk | Low risk and high risk | Low risk | Low risk |
| H.-K. Son2010 | Low risk | Low risk | Some concerns | Low risk and some concerns | Low risk and some concerns | Low risk | Low risk |
| Ki-Su Kim2014 | Low risk | Low risk | Some concerns | Low risk | Low risk and high risk | Low risk | Low risk |
| Geng Zong2018 | Low risk | Low risk | Some concerns | Low risk and some concerns | Low risk and some concerns | Low risk | Low risk |
| Andreas Tornevi2019 | Low risk | Low risk | Some concerns | Low risk | Low risk and some concerns | Low risk | Low risk |
| Lindsay M. Jaacks2019 | Low risk | Low risk | Some concerns | Low risk and some concerns | Low risk and some concerns | Low risk | Low risk |
| Xu Han2020 | Low risk | Low risk | Some concerns | Low risk | Low risk and some concerns | Low risk | Low risk |
| El Hadia Mansouri2021 | Low risk | Low risk | Some concerns | Low risk | Low risk and some concerns | Low risk | Low risk |
| Dianna J. Magliano2021 | Low risk | Low risk | Some concerns | Low risk | Low risk and some concerns | Low risk | High risk |
| Neha Tawar2022 | Low risk | Low risk and some concerns | Some concerns | Low risk and some concerns | Low risk and some concerns | Low risk | Low risk |
| Dolley Charles2021 | Low risk | Low risk | Some concerns | Low risk | Low risk and some concerns | Low risk | Low risk |
| Chengyong Jia2023 | Low risk | Low risk | Some concerns | Low risk | Low risk and high risk | Low risk | Low risk |
| Jiayu Shi2024 | Low risk | Low risk | Some concerns | Low risk and some concerns | Low risk and some concerns | Low risk | Low risk |

**Supplementary Table 3. Search strategies for several major databases**

| Database | Keywords | Operators | Final search query |
| --- | --- | --- | --- |
| Web of science | 2,2-bis (4-Chlorophenyl)-1,1,1-trichloroethane , dichlorodiphenyltrichloroethane, dichlorodiphenyldichloroethylene, dichlorodiphenyldichloroethane, DDT , DDTs, DDE, DDD, *p,p*′-DDT, *o,p*′-DDT, *p,p*′-DDD, *o,p*′-DDD , *p,p*′-DDE, *o,p*′-DDE, Type 2 diabetes mellitus, T2DM, T2D. | Or, and, (), [] | (2,2-bis [4-Chlorophenyl]-1,1,1-trichloroethane or dichlorodiphenyltrichloroethane or dichlorodiphenyldichloroethylene or dichlorodiphenyldichloroethane or DDT or DDTs or DDE or DDD or *p,p*′-DDT or *o,p*′-DDT or *p,p*′-DDD or *o,p*′-DDD or *p,p*′-DDE or *o,p*′-DDE) and (Type 2 diabetes mellitus or T2DM or T2D). |
| Embase | 2,2-bis (4-Chlorophenyl)-1,1,1-trichloroethane , dichlorodiphenyltrichloroethane, dichlorodiphenyldichloroethylene, dichlorodiphenyldichloroethane, DDT , DDTs, DDE, DDD, *p,p*′-DDT, *o,p*′-DDT, *p,p*′-DDD, *o,p*′-DDD , *p,p*′-DDE, *o,p*′-DDE, Type 2 diabetes mellitus, T2DM, T2D. | Or, and, (), [] | (2,2-bis [4-Chlorophenyl]-1,1,1-trichloroethane or dichlorodiphenyltrichloroethane or dichlorodiphenyldichloroethylene or dichlorodiphenyldichloroethane or DDT or DDTs or DDE or DDD or *p,p*′-DDT or *o,p*′-DDT or *p,p*′-DDD or *o,p*′-DDD or *p,p*′-DDE or *o,p*′-DDE) and (Type 2 diabetes mellitus or T2DM or T2D). |

**Supplementary Table 3. Cont**

| Database | Keywords | Operators | Final search query |
| --- | --- | --- | --- |
| PubMed | 2,2-bis (4-Chlorophenyl)-1,1,1-trichloroethane , dichlorodiphenyltrichloroethane, dichlorodiphenyldichloroethylene, dichlorodiphenyldichloroethane, DDT , DDTs, DDE, DDD, *p,p*′-DDT, *o,p*′-DDT, *p,p*′-DDD, *o,p*′-DDD , *p,p*′-DDE, *o,p*′-DDE, Type 2 diabetes mellitus, T2DM, T2D. | Or, and, (), [] | (2,2-bis [4-Chlorophenyl]-1,1,1-trichloroethane or dichlorodiphenyltrichloroethane or dichlorodiphenyldichloroethylene or dichlorodiphenyldichloroethane or DDT or DDTs or DDE or DDD or *p,p*′-DDT or *o,p*′-DDT or *p,p*′-DDD or *o,p*′-DDD or *p,p*′-DDE or *o,p*′-DDE) and (Type 2 diabetes mellitus or T2DM or T2D). |

**Supplementary Table 4. The main mechanisms and dosage range of DDT and its metabolites affecting the occurrence of T2DM**

| **Reference** | **Journal** | **Species** | **Principal mechanism** | **Range doses** | **T2DM development** |
| --- | --- | --- | --- | --- | --- |
| Jing Zhang et al 2024 | Toxicology Research | Mouse | Down-regulated insulin receptor sub­  strate (IRS)-2 mRNA expression | LD50 = 2170 mg·kg^−1^ | Insulin resistance Risk of diabetes |
| Lasram et al 2024 | [Toxicology](https://www.sciencedirect.com/journal/toxicology) | Mouse | Induced inflammation and oxidative stress resulting in  inactivation of IRS-1 | 6mg·kg^−1^ | Insulin resistance Risk of diabetes |
| Kim et al 2014 | J Biochem Mol Toxicol | Cell | Alter key regulators of adipose tissue lipid  metabolism and alter the expression of key mediators in the insulin  signaling pathway | 10-20uM | Hyperglycaemia  Risk of diabetes |
| Czajka et al 2019 | [Environmental Research](https://www.sciencedirect.com/journal/environmental-research) | Mouse | Induce oxidative stress and damage mitochondrial compartments | 1.6mg·kg^−1^ | Hyperglycemia  Insulin resistance |
| Ibrahim et al 2011 | PLOS One | Mouse | Impaired glucose uptake | NA | Insulin resistance |
| Turyk  et al 2009 | Environmental Health Perspectives | Human | Reduced glucose uptake | 2.3-49.2ng | Insulin resistance  Risk of diabetes |
| Ruzzin et al 2010 | Environmental Health Perspectives | Cell | Reduced glucose uptake | 1nM-100nM | Insulin resistance |
| [Sarah I Daniels](javascript:;) et al 2018 | Journal of the Endocrine Society | Human | Reduced  insulin sensitivity and glucose metabolic dysregulation | 0.11-3.82ng·ml^−1^ | Insulin resistance |
| . Pavlikova N et al 2015 | [Environmental Research](https://www.sciencedirect.com/journal/environmental-research) | Cell | Reduced protein expression of genes  associated with hyperglycemic stress response | O.1nM-100uM | Hyperglycaemia |
| Jérôme Ruzzin et al 2010 | [Environmental Research](https://www.sciencedirect.com/journal/environmental-research) | Mouse | Induced down-regulation of insulin-induced gene-1  (Insig-1) and Lpin1 | 1nM-100nM | Insulin resistance |
